# UHPLC-MS/MS-Based Metabolomics Identifies Freshness Biomarkers and Temporal Spoilage Threshold in Refrigerated Goose Meat

**DOI:** 10.3390/foods14172950

**Published:** 2025-08-24

**Authors:** Wen Gao, Zhengfeng Cao, Qiang Bao, Qingping Tang, Zhu Bu, Guohong Chen, Bichun Li, Qi Xu

**Affiliations:** 1 Jiangsu Institute of Poultry Science, Yangzhou 225125, China; dx120190117@yzu.edu.cn (W.G.); tqp0979@163.com (Q.T.); jsbuzhu@163.com (Z.B.); 2 Key Laboratory for Evaluation and Utilization of Poultry Genetic Resources of Ministry of Agriculture and Rural Affairs, Yangzhou University, Yangzhou 225009, China; dx120200144@stu.yzu.edu.cn (Q.B.); ghchen2019@yzu.edu.cn (G.C.); yubcli@yzu.edu.cn (B.L.); 3 Joint International Research Laboratory of Agriculture and Agri-Product Safety, The Ministry of Education of China, Yangzhou University, Yangzhou 225009, China

**Keywords:** refrigerated goose meat, UHPLC-MS/MS, widely targeted metabolomics, metabolic biomarker, freshness

## Abstract

The dynamic metabolic landscape underlying goose meat quality deterioration during refrigerated storage remains incompletely elucidated. Here, ultra-high performance liquid chromatography-tandem mass spectrometry (UHPLC-MS/MS)-based widely targeted metabolomics was employed to characterize metabolic profiling in refrigerated goose meat. Orthogonal partial least squares discriminant analysis (OPLS-DA) revealed 211 differential metabolites, while random forest regression (RFR) identified 30 candidate biomarkers. Seven metabolites, including xanthine, oxidized glutathione, and inosine 5′-monophosphate, exhibited significant correlations with total volatile basic nitrogen (TVB-N). By integrating potential biomarkers, metabolic pathways involving purines, amino acids, and sugars were identified as underlying mechanisms of goose meat spoilage. Notably, through comprehensive analysis of time-dependent correlations between physicochemical properties and metabolic profiles, a temporal threshold for quality deterioration in refrigerated goose meat was identified as day 5. These findings deepen our understanding of metabolite variations in refrigerated goose meat and provide a basis for optimizing storage protocols. The identified biomarkers may enable rapid detection kits and smart packaging systems for poultry industry applications.

## 1. Introduction

The goose industry holds a pivotal position within China’s waterfowl sector, constituting a vital component of global poultry production. According to statistics asserted by Food and Agriculture Organization (FAO), the number of commercial geese in China reached 522 million in 2023, accounting for about 95% of global output. Goose meat is nutritionally distinguished by its high protein content (∼20%) with complete essential amino acids, abundant minerals (Fe, Zn, Mg, K, P, Ca, Cu, Na), and B-complex vitamins. Its lipid profile exhibits nutritional advantages, characterized by moderate total fat (3–4%), high proportion of unsaturated fatty acids (∼70%), elevated linolenic acid levels, and relatively low cholesterol (52–76 mg/100g fresh weight) compared to conventional meats [1,2]. However, these distinctive attributes, particularly the high unsaturation of lipids and active endogenous enzyme systems, render goose meat highly susceptible to quality deterioration during postmortem handling, posing significant challenges to its preservation and commercialization.

Postmortem quality deterioration of meat involves complex biochemical processes driven by the interplay of autolytic enzymatic reactions, lipid oxidation, and microbial metabolism [3]. Autolytic degradation initiates with ATP dephosphorylation, generating flavor-enhancing inosine monophosphate (IMP) that progressively degrades to inosine (HxR) and hypoxanthine (Hx) through the action of endogenous nucleases and phosphatases. These metabolites are strongly correlated with freshness loss, serving as critical indicators of meat spoilage [4]. Concurrently, lipid oxidation, particularly of polyunsaturated fatty acids (PUFAs), proceeds via free radical chain reactions, producing volatile aldehydes (e.g., hexanal, nonanal) and ketones that are responsible for rancid off-odors and off-flavors [5]. Microbial proliferation, dominated by psychrotrophic bacteria such as *Pseudomonas* and *Acinetobacter,* further accelerates spoilage through proteolytic activity and amino acid decarboxylation [6]. This latter process generates biogenic amines, including histamine (a potential toxin), cadaverine, and tyramine, which not only reflect quality deterioration but also pose food safety risks [7]. The dynamic balance and interaction between these pathways ultimately determine the shelf-life of goose meat, yet the specific mechanisms governing these processes in goose meat remain poorly characterized, despite its distinct biochemical composition differing from other poultry species.

Metabolomic approaches, leveraging advanced analytical techniques, such as mass spectrometry, have emerged as powerful tools for deciphering spoilage biochemistry through comprehensive profiling of low-molecular-weight metabolites (<1500 Da). In chicken, metabolomic studies have identified IMP and Hx as reliable freshness markers [8], while in duck, metabolomic analyses revealed the accumulation of putrescine and cadaverine as key indicators of storage-induced quality decline [9]. Research on pork and fish has further established robust correlations between lipid oxidation products (e.g., malondialdehyde) and sensory deterioration, clarifying the metabolic pathways underlying quality changes [10]. Nevertheless, existing research overlooks goose meat, a significant omission given its commercial value in Asian markets and unique physiological traits. The high PUFA content in goose meat [2], combined with distinctive postmortem glycogen metabolism and enzyme activity patterns, likely accelerates spoilage kinetics through enhanced oxidative reactions and increased availability of microbial substrates. Moreover, the ongoing shift in the industry from live poultry markets to chilled products necessitates precise shelf-life prediction, a capability currently limited by the lack of comprehensive metabolic baselines for goose meat.

In this study, ultra-high performance liquid chromatography-tandem mass spectrometry (UHPLC-MS/MS) was implemented to characterize temporal metabolic dynamics in refrigerated goose meat under 4 °C preservation conditions (0, 3, 5, and 7 days), while multivariate statistical analysis was employed to identify critical freshness-associated biomarkers and pathways. Furthermore, through systematic investigation of the dynamic correlations between physicochemical properties and metabolic profiles under refrigerated storage, a temporal threshold for quality deterioration was identified in Yangzhou goose meat. The results not only offer new insights into the further understanding of the variation in goose metabolite profiles during storage but also provides a scientific basis for better quality control and storage efficiency of poultry meat.

## 2. Materials and Methods

### 2.1. Bird Handling

All procedures for goose care and use were approved by and conducted in accordance with guidelines from the Institutional Animal Care and Use Committee of Yangzhou University (approval ID: YZUDWSY2021-16). One-day old purebred Yangzhou geese (*Anser cygnoides*) were reared in the farm of Tiange Goose Industry Co. Ltd. (Yangzhou, Jiangsu, China) under controlled conditions, with free access to water and feed, until the 4th week. The temperature was approximately maintained at 32 °C using a controlled heater, and was gradually reduced for comfort. On day 28, a total of 120 healthy female goslings with similar body weight were selected and kept in a pen with a playground and pool. The geese were exposed to natural light and temperature. Basal diets were formulated according to the national standards of R.P. China-Yangzhou goose (GB/T 36784-2018) [11], for which the ingredients and chemical composition are shown in Table 1.

### 2.2. Meat Sampling and Storage

A total of 24 female Yangzhou geese (70-day-old) were randomly selected and fasted for 12 h before slaughter, with free access to water. Each goose was anesthetized with 0.1 mL/kg xylazine hydrochloride (Shengda, Changchun, China) before being sacrificed by jugular puncture. Immediately after slaughter, the breast muscle was aseptically excised from each carcass. Each individual breast muscle sample was placed into a separate, sterile, low-density polyethylene (LDPE) bag. The bags were heat-sealed to ensure containment but were not vacuum-packed or flushed with inert gas, thus allowing exposure to ambient air within the bag headspace during storage. All samples were transported to the laboratory immediately under refrigeration (4 °C). Upon arrival, samples were aseptically divided into four treatment groups (FM, 3DM, 5DM, and 7DM) based on the designated storage time at 4 °C (0, 3, 5, and 7 days, respectively). Each group consisted of six replicate samples (one breast from each of six geese). Each replicate sample remained in its individually sealed LDPE bag and was stored statically at 4 °C for the designated duration.

### 2.3. Meat Quality Measurements

Breast meat samples stored for 0 day, 3 days, 5 days, and 7 days were selected for meat quality measurements (color, pH, shear force, water-holding capacity (WHC), moisture, protein, intramuscular fat (IMF), collagen content, and total volatile basic nitrogen (TVB-N)).

The color of breast muscle was measured using a Minolta CR-400 colorimeter (Konika Minolta, Tokyo, Japan) with a D65 light source and 10° standard observer angle. The instrument provided information about the lightness (L* value), redness (a* value), and yellowness (b* value) of the muscle samples. Each attribute was measured in triplicate for each sample. The pH value of breast muscle was measured at a depth of 10 mm into the muscle, using a pH meter (DELTA 320, Mettler Toledo, Columbus, OH, USA). The pH meter was standardized by a two-point calibration method with commercially available standard buffer solutions of pH 4.0 and pH 7.0. Cooking loss was used to assess water-holding capacity (WHC) following the method described by Xing et al. [12]. Briefly, meat samples were cut and packed into cooking bags and heated at 80 °C until a core temperature of 72 °C was reached. The samples were cooled to room temperature and reweighed to measure cooking loss. Subsequently, the shear force of samples was determined using a digital tenderness meter (C-LM3B, Tenovo, Beijing, China). Muscle samples with dimensions of 1.0 cm × 1.0 cm × 3.0 cm (width × height × length), cut orthogonal to the fiber direction, were used for the measurement. The proximate composition, including moisture, protein, IMF, and collagen, was analyzed using a FoodScanTM Meat Analyzer (FOSS, FoodScan 78800, Hilleroed, Denmark). Following all exterior fat, connective tissue, muscle membrane, and tendon removal, meat samples were cut into small pieces and coarse ground. Then, 180 g ground samples were placed in a 140 mm disc for evaluation by FoodScan [13].

The TVB-N content of muscle samples during storage was determined by the Kjeldahl method using an automatic Kjeldahl nitrogen analyzer (K9840, Hannon Instruments, Jinan, China), referring to the Chinese National Food Safety Standard (GB 5009.228-2016) [14]. Briefly, 10 g (±0.1 g) of muscle sample were homogenized with 75 mL of cold distilled water. After equilibrating for 30 min, the sample was filtrated using NO. 101 filter paper (Advantec, Tokyo, Japan). A 10-mL aliquot of filtrate was added to the distillation tube, followed by 10 mL magnesia (10 g/L), and distilled for 5 min. Meanwhile, 10 mL of distilled water was set as the blank control. Then, the distillate was collected in an Erlenmeyer flask containing 20 mL of 20 g/L boric acid with methyl red-bromocresol green mixed indicator and titrated with 0.01 mol/L hydrochloric acid solution. The TVB-N content was calculated using the following equation:

(1) $$TVB-N(\mathrm{mg}/100\;g)=\frac{(V_{1}-V_{2})\times c\times 14}{m}\times 100$$

where *V*_1_ represents the titration volume of the tested sample (mL); *V*_2_ represents the titration volume of the blank control (mL); c represents the concentration of hydrochloric acid (mol/L); m represents the weight of the tested sample (g). Each analysis was repeated in triplicates.

### 2.4. Metabolite Extraction

Each breast muscle tissue sample was homogenized 4 times in an Eppendorf tube containing a steel ball at 30 Hz for 30 s, and 1 mL 70% methanol was added. The samples were oscillated for 5 min and then placed in an ice-bath for 15 min. After that, the samples were centrifuged at 12,000 rpm at 4 °C for 10 min, and 400 μL of the supernatant was placed into a new Eppendorf tube and stored in a −20 °C freezer overnight. The supernatant was then centrifuged at 12,000 rpm at 4 °C for 3 min, and 200 μL of the supernatant in the liner of the corresponding injection bottle was collected for on-board analysis. Quality control (QC) samples were prepared by mixing an equal aliquot of the supernatants from all of the samples to correct the deviation of the analytical results of the mixed sample and the errors caused by the analytical instrument.

### 2.5. Ultra Performance Liquid Chromatography Conditions

The sample extracts were analyzed using an LC-ESI-MS/MS system (UPLC, ExionLC AD, http://sciex.com.cn/; MS, QTRAP○R System, http://sciex.com/). The analytical conditions were as follows, UPLC: column, Waters ACQUITY UPLC HSS T3 C18 (1.8 μm, 2.1 mm × 100 mm); column temperature, 40 °C; flow rate, 0.4 mL/min; injection volume, 2 μL; solvent system, water (0.1% formic acid); acetonitrile (0.1% formic acid); gradient program, 95:5 *V*/*V* at 0 min, 10:90 *V*/*V* at 10.0 min, 10:90 *V*/*V* at 11.1 min, 95:5 *V*/*V* at 14.0 min.

### 2.6. ESI-QTRAP-MS/MS

LIT and triple quadrupole (QQQ) scans were acquired on a triple quadrupole-linear ion trap mass spectrometer (QTRAP, SCIEX, Toronto, ON, Canada), QTRAP○RLC-MS/MS System, equipped with an ESI Turbo Ion-Spray interface, operating in positive and negative ion modes and controlled by Analyst 1.6.3 software (Sciex). The ESI source operation parameters were as follows: source temperature of 500 °C; ion spray voltage (IS) 5500 V (positive), −4500 V (negative); ion source gas Ⅰ (GSⅠ), gas Ⅱ (GSⅡ), curtain gas (CUR) were set at 55, 60, and 25.0 psi, respectively; the collision gas (CAD) was high. Instrument turning and mass calibration were performed with 10 and 100 μmol/L polypropylene glycol solution in QQQ and LIT modes, respectively. A specific set of MRM transitions were monitored for each period according to the metabolites eluted within this period.

### 2.7. Metabolite Identification and Classification of Targeted Classes

Metabolites were identified by matching with reference standards for retention time and MS/MS fragmentation patterns and aligning with the self-built Metware Biotechnology database (MWDB) using accurate molecular weight (mass error < 10 ppm) and characteristic ion transitions. As described in Section 2.8, identified metabolites were further annotated using the KEGG Compound database (http://www.kegg.jp/kegg/compound/) and mapped to the KEGG Pathway database for functional analysis.

According to their chemical structures, biological functions, and relevance to meat freshness and spoilage, the identified metabolites were classified into targeted classes, including amino acids and their metabolites, nucleotides and their metabolites, lipid metabolites, carboxylic acids and their metabolites, organic acids and their metabolites, alcohol and amines, coenzymes, and vitamins. These targeted classes were selected for their direct relevance to characterizing metabolic shifts underlying freshness deterioration, flavor changes, and oxidative damage during refrigerated storage of goose meat.

### 2.8. Statistical Analysis

All experiments were conducted with six biological replicates. For meat quality measurements (color, pH, shear force, WHC, moisture, protein, IMF, collagen content, and TVB-N), each analysis was conducted in triplicate. The comparison among means was evaluated by performing one-way ANOVA with Duncan’s multiple range tests at a significance level of 0.05. The results are expressed as the mean ± SEM and visualized by GraphPad Prism 9 software (GraphPad Software, San Diego, CA, USA). Pearson’s correlation coefficient between TVB-N and differential metabolites was analyzed using IBM SPSS Statistics 27 (SPSS Inc., Chicago, IL, USA) and visualized in Origin 2024 software (OriginLab Corp., Northampton, MA, USA).

Unsupervised PCA (principal component analysis) was performed by statistics function prcomp within R (https://www.r-project.org) to access the metabolite diversity between and within group samples. The HCA (hierarchical cluster analysis) results of samples and metabolites were presented as heatmaps with dendrograms, while Pearson correlation coefficients (PCC) between samples were calculated by the cor function in R and presented as only heatmaps. Significantly regulated metabolites between groups were determined by VIP (variable importance in projection) ≥1 and absolute Log_2_FC (fold change) ≥1. VIP values were extracted from orthogonal partial least squares discriminant analysis (OPLS-DA) results, which also contain score plots and permutation plots, and generated using R package MetaboAnaystR (version 4.0). The potential biomarkers were first analyzed using random forest via Metware Cloud, a free online data analysis platform (https://cloud.metware.cn). Subsequently, differences among multiple groups for these biomarkers were analyzed using the Kruskal-Wallis rank sum test. Identified metabolites were annotated using KEGG Compound database (http//www.kegg.jp/kegg/compound/), annotated metabolites were then mapped to KEGG PathwLllllay database (http://www.kegg.jp/kegg/pathway.html). Pathways with significantly regulated metabolites mapped to them were then fed into MSEA (metabolite set enrichment analysis), and their significance was determined by hypergeometric test *p*-values.

## 3. Results and Discussion

### 3.1. Meat Quality Attributes

**Figure 1:**
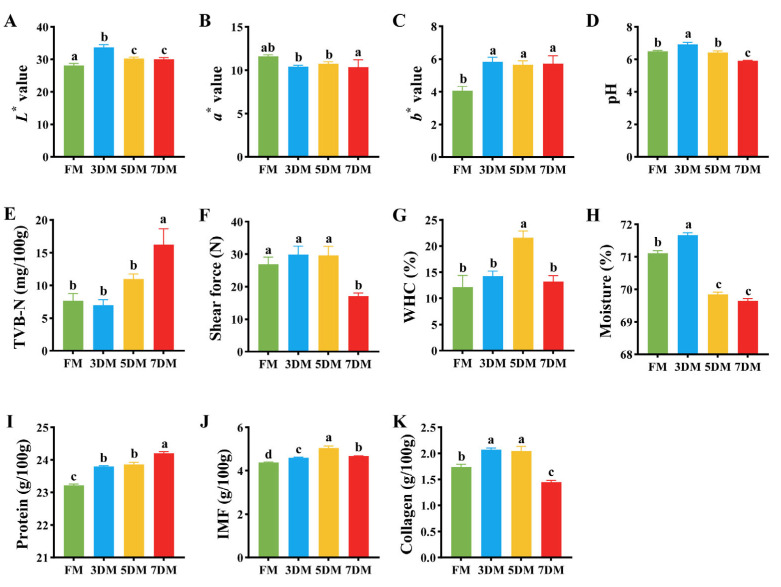
Dynamics of physicochemical properties in refrigerated goose meat. (**A**–**K**) Temporal changes in color parameters (L*, a*, b*), pH, TVB-N, texture characteristics (shear force, WHC), and proximate composition (moisture, protein, IMF, collagen) during 7-day storage at 4 °C. FM: Fresh meat (0 day); 3DM/5DM/7DM: Days of storage. Different superscripts (a–d) denote significant differences (*p* < 0.05).

Color serves as an intuitive meat quality indicator influencing consumers’ purchasing decisions, with the lightness (L*), redness (a*), and yellowness (b*) values being important parameters associated with the acceptance of meat color [15]. The L* value, functioning as a primary metric for gauging meat paleness, has been reported to exhibit a negative correlation with the myoglobin (Mb) content. Here, goose breast meat exhibited a significant increase in L* value during refrigeration, reaching its peak on the third day (Figure 1A). This result suggested substantial Mb degradation during refrigeration, with the most pronounced decrease occurring within the first three days, which might be associated with Mb oxidation [16]. A significant reduction in a* value (*p* < 0.05) was observed over the first 3 days of refrigeration prior to stabilization, whereas the b* value which demonstrated a marked increase during the same period before reaching equilibrium (Figure 1B,C). Previous evidence has demonstrated that the decrease in a* value can be attributed to the oxidation and the formation of metmyoglobin (MetMb), while the change in the b* is primarily related to yellow pigment formed by the reaction between lipids and amine during refrigeration [17,18].

The pH value is an important parameter for assessing meat quality, as it directly affects color stability, water-holding capacity, and microbial activity through its influence on protein denaturation and postmortem metabolic processes [19]. As shown in Figure 1D, the pH value significantly increased during the initial 3 days of refrigeration (*p* < 0.05), returned to the baseline level by day 5, and subsequently declined below the initial value by day 7 (*p* < 0.05). The initial pH increase aligns with typical postmortem proteolytic deamination by endogenous enzymes and spoilage bacteria (e.g., *Pseudomonas*), generating alkaline compounds like ammonia and amines [6]. However, the subsequent pH decline observed in goose meat might be attributed to the growth of lactic acid bacteria (LAB) and the accumulation of acidic metabolites. As Calo-Mata et al. (2008) demonstrated, LAB actively reduce muscle pH through lactic acid production, thereby inhibiting other microorganisms [20]. This acidification is further driven by the accumulation of lactic acid from anaerobic sugar fermentation, phosphoric acid from ATP catabolism, and the dissolution of CO_2_ in the muscle tissue, consistent with mechanisms reported for fish by Li et al. (2016) [21]. Notably, the reduced pH alters meat’s physical properties by increasing light scattering and modifying the transverse refractive index of myofilaments, ultimately inducing pale coloration [22]. Moreover, the pH decline could also induce structural changes in myofibrillar proteins during refrigerated storage. As evidenced by Alvarado et al. (2004), an early postmortem pH reduction under chilling conditions might induce alterations in myofibrillar proteins in the turkey pectoralis muscle, such as reduced gel strength, increased cook loss, and enhanced precipitation of phosphorylase onto myofibrils, which are closely associated with the development of pale, soft, and exudative tissues [23].

TVB-N, an internationally standardized biochemical indicator of meat freshness, is primarily generated through microbial decomposition and enzymatic degradation of proteins and non-protein nitrogenous compounds during storage [1]. As shown in Figure 1E, a progressive increase was observed in the TVB-N content during refrigeration. The TVB-N values remained relatively low within the first 3 days, increased to approximately 10 mg/100g on the 5th day, and then rose sharply to exceed 15 mg/100g on the 7th day (*p* < 0.05). According to the Chinese national food safety standard for fresh and frozen poultry meat (GB2710-1996) [24], the TVB-N threshold is established at 20 mg/100 g for qualified products. Notably, duck and goose meat classified as “first-class freshness” exhibited stricter TVB-N levels of ≤13 mg/100g (GB10148-88) [25]. Our data suggested that the TVB-N content exhibited a time-dependent increase throughout refrigerated storage, with goose breast meat maintaining acceptable quality parameters within the 7 days of refrigeration. Notably, day 5 of refrigeration emerged as the critical threshold for quality deterioration, marked by accelerated biochemical changes in meat components.

To investigate the textural deterioration that occurs during the refrigeration process of goose meat, shear force, water-holding capacity (WHC), and moisture content were analyzed as key structural parameters. As shown in Figure 1F, no significant difference was observed in the shear force of goose meat during the first 5 days of refrigeration, whereas a highly significant decrease occurred on day 7 (*p* < 0.05), indicating that a critical transition in tenderness occurred specifically with 5–7 days of refrigeration. This observation aligns with established evidence that prolonged refrigeration activates endogenous proteolytic enzymes (e.g., cathepsins and trypsin-like proteases), which systematically degrade key structural proteins including actin and myosin, thereby disrupting the Z-disc architecture and myofibrillar integrity [26]. These structural alterations led to disorganized muscle fiber arrangement and the observed significant decrease in shear force. The observed increase in WHC on day 5 of refrigeration, followed by a return to baseline level (Figure 1G), may be attributed to dynamic structural changes in muscle proteins during early cold storage [27]. Initially, proteolytic enzyme activity likely degraded myofibrillar proteins, exposing hydrophilic groups and temporarily enhancing WHC. This transient WHC elevation coincides with the marked moisture content reduction in the first 5 days (Figure 1H), possibly due to extracellular fluid expulsion from muscle contraction and surface dehydration [28]. The subsequent WHC decline after day 5 might reflect progressive protein denaturation and microstructural damage, accompanied by disrupted myofiber integrity during prolonged storage. Moisture stabilization from day 5–7 could indicate equilibrium between continued intracellular water migration and structural collapse limiting further fluid loss [29].

To characterize the dynamic changes in the chemical composition of goose meat during refrigeration, we subsequently determined the protein, intramuscular fat (IMF), and collagen contents. The protein content increased significantly during refrigerated storage from day 0 to day 7, with notable rises observed between days 0 and 5 and days 5 and 7 (*p* < 0.05) (Figure 1I). This phenomenon could be attributed to the proteolytic degradation of myofibrillar proteins into soluble peptides and amino acids, coupled with the concentration of protein fractions resulting from water loss throughout the storage duration [30]. The IMF content also exhibited a marked rise from 0 to 7 days, reaching its highest level on day 5 (*p* < 0.05) (Figure 1J). This elevation in IMF likely stemmed from moisture evaporation under refrigeration conditions, resulting in a relative increase in lipid concentration. The collagen content displayed a biphasic pattern, increasing until day 5 before declining significantly by day 7 (*p* < 0.05) (Figure 1K). The initial elevation may result from the sustained activity of endogenous proteases degrading collagen fibers into smaller peptides or soluble fractions, which amplifies detectable collagen levels [31]. However, the subsequent reduction at day 7 suggested progressive collagen degradation by endogenous enzymes in muscle such as collagenase, consistent with findings in yak meat [32]. These results indicate that refrigerated storage duration significantly impacts the quality of goose breast meat, with day 5 emerging as a critical threshold for key physicochemical and sensory attributes associated with meat deterioration.

### 3.2. Quality Control and PCA of Components in Refrigerated Goose Meat

**Figure 2:**
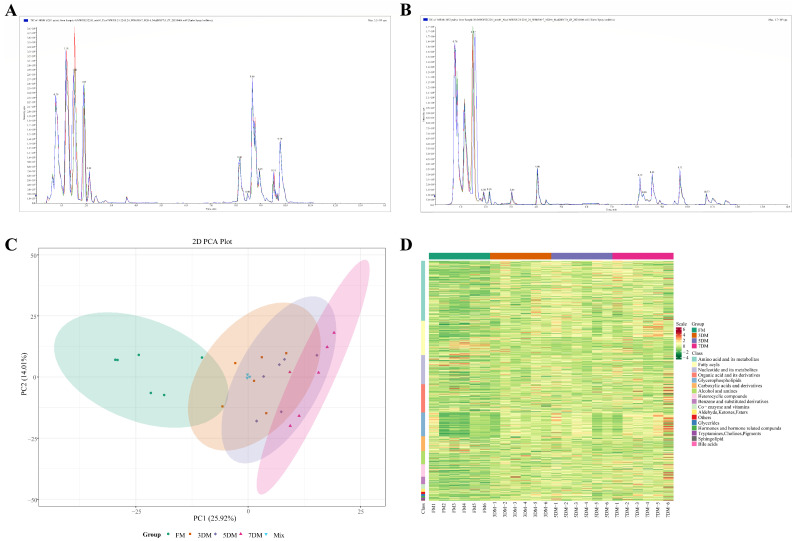
Quality control and metabolic profile analysis of refrigerated goose samples. (**A**) Positive-ionization-mode total ion current spectra of three quality control samples. Different colors represent samples. (**B**) Negative-ionization-mode total ion current spectra of three quality control samples. Different colors represent samples. (**C**) Principal component analysis (PCA) of all samples in positive and negative ion modes among FM, 3DM, 5DM, 7DM, and Mix. Different colors areas represent the confidence intervals of different groups. (**D**) Heat map demonstrating the expression pattern of all detected peaks in positive and negative ion modes among FM, 3DM, 5DM, and 7DM.

Total ion current (TIC) chromatograms were obtained based on the positive and negative ion modes (Figure 2A,B). The horizontal coordinate represents the retention time for metabolite detection, and the vertical coordinate represents the intensity of ion flow. The overlapping peaks indicated high stability of the detection system and excellent repeatability of metabolite extraction and detection. Unsupervised PCA (principal component analysis) results of 27 mixed samples (including 3 QC samples) were obtained based on both positive and negative ion mode data (Figure 2C). The PCA score plot displayed a tight clustering of QC samples, suggesting that actual biological differences contributed to the separation among samples, rather than instrumental drift occurring throughout the analysis. A partial overlap in the confidence intervals for the metabolites in fresh goose meat (FM) and meat refrigerated for 3 days (3DM) was observed, while, a significant overlap in the confidence intervals was shown in goose meat which had been refrigerated for 3, 5, and 7 days (3DM, 5DM, 7DM), indicating that the metabolites in refrigerated goose meat changed to varying degrees over time, which may be caused by decomposition during storage. Additionally, the expression pattern of all detected peaks was depicted by a heat map (Figure 2D).

### 3.3. Screening and Identifying Differential Metabolites

Metabolite identification and quantification were performed based on the self-built database MWDB (Metware Biotechnology Co., Ltd. Wuhan, China) and MRM (multiple reaction monitoring) of triple quadrupole (QQQ) scans. A total of 748 metabolites were annotated, including 41 alcohol and amines, 9 aldehyde, ketones, and esters, 187 amino acids and their metabolites, 23 benzene and substituted derivatives, 1 bile acid, 46 carboxylic acids and their derivatives, 15 coenzyme vitamins, 107 fatty acyls, 6 glycerolipids, 74 glycerophospholipids, 39 heterocyclic compounds, 5 hormones and their related compounds, 91 nucleotides and their metabolites, 88 organic acids and their metabolites, 4 sphingolipids, 5 tryptamines, cholines, and pigments, and 7 others (Table S1).

**Figure 3:**
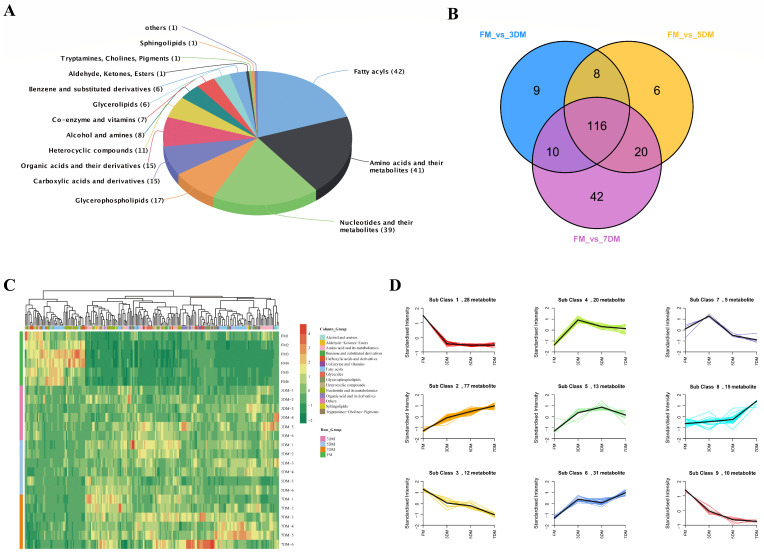
Classification of differential metabolites in refrigerated goose breast meat. (**A**) Classification of 211 differential metabolites. The value in each bracket indicates the number of differential metabolites. (**B**) Venn diagram of the differential metabolites. (**C**) Heat map revealing the expression pattern of 211 differential metabolites in four groups (FM, 3DM, 5DM, 7DM). (**D**) The K-means clustering result of differential metabolites in four groups (FM, 3DM, 5DM, 7DM).

To further screen the differential metabolites associated with the freshness of chilled goose meat, OPLS-DA was performed to determine the significantly regulated metabolites between groups by the variable importance of the projection (VIP) values. The score plots and permutation plots are presented in Figure S1. All models demonstrated strong predictive validity, with Q^2^ values ranging from 0.881 to 0.929 (*p* < 0.005). The fold change was obtained by dividing the average abundance of the metabolites between groups. A total of 211 differential metabolites combined of 15 classes were identified by defining the fold change ≥2 (upregulated) or ≤0.5 (downregulated) and VIP ≥1 (Table S2). Fatty acyls accounted for the most proportion of total differential metabolites (19.91%), followed by amino acids and their metabolites (19.43%), nucleotides and their metabolites (18.48%), glycerophospholipids (8.06%), carboxylic acids and derivatives (7.11%), organic acids and their metabolites (7.11%), etc. (Figure 3A). Notably, in comparison with the downregulated metabolites, more upregulated metabolites were observed in each group, which simultaneously showed relative accretion over time (Table S2). Likewise, as the storage time was prolonged, an increase in differential metabolites compared to the fresh refrigerated goose meat was also found via the Venn diagram (Figure 3B). Meanwhile, 116 differential metabolites were identified as the common metabolites among the three groups (FM vs. 3DM, FM vs. 5DM, and FM vs. 7DM), which may play indispensable roles in the spoilage of goose meat during storage. The clustering heat map of 211 differential metabolites delineated the relative abundance of these annotated metabolites in different groups, showing trends in metabolic profiles consistent with the PCA (Figure 3C).

To further cluster the differential metabolites with similar metabolic profiles during refrigerated storage, the data were normalized and centralized for K-means clustering. As shown in Figure 3D, 9 classes were characterized, with the overall trend of 156 differential metabolites increasing in classes 2, 4, 5, 6, and 8, and 55 differential metabolites decreasing over time in classes 1, 3, 7, and 9. Of note, 77 metabolites in class 2 kept rising with storage time, while 22 metabolites in classes 3 and 9 exhibited sustained reductions over time. In class 1, 28 metabolites consisting of nucleotides and their metabolomics, organic acids and their derivatives, carboxylic acids and derivatives, and fatty acyls showed a sharp decline during the first 3 days of refrigeration but remained stable thereafter. In stark contrast, the quantity of 33 metabolites in classes 4 and 5, mainly belonging to amino acids and their metabolites, fatty acyls, nucleotides and their metabolites, alcohol and amines, as well as glycerophospholipids, increased dramatically from day 0 to day 3 but remained relatively stable thereafter. Overall, these results suggest that metabolites related to the freshness of goose meat showed different metabolic patterns during refrigeration, and most of the metabolites were upregulated with storage time.

### 3.4. Screening Biomarkers Related to Freshness in Refrigerated Goose Meat

**Figure 4:**
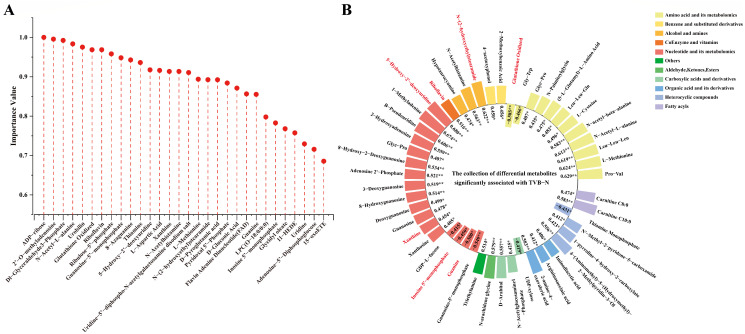
Identification of spoilage-related biomarkers in refrigerated goose meat using random forest regression. (**A**) Dot plot representing the top 30 metabolic biomarkers identified by random forest regression. (**B**) Radial distribution of 49 differential metabolites significantly associated with TVB-N accumulation in refrigerated goose meat. Metabolites labeled in red are among the top 30 biomarkers screened by random forest regression. The numerical values indicate Pearson correlation coefficients calculated between differential metabolites and TVB-N contents. * *p* < 0.05 and ** *p* < 0.01 indicate significant and extremely significant difference, respectively.

Random forest regression (RFR), a supervised learning algorithm based on an ensemble of decision trees [33], was applied to identify metabolic biomarkers associated with the freshness of goose meat during refrigerated storage. Potential metabolic biomarkers were selected by the varSeIRF package in R, using a backward elimination procedure with standard implementation of the randomForest package [34]. Through regression analysis of metabolite relative peak areas against storage duration to determine variable importance values, the top 30 metabolites were ultimately identified as potential metabolic biomarkers associated with goose meat freshness during refrigeration, comprising 17 upregulated and 13 downregulated biomarkers (Figure 4A and Figure S2, Table S3). To further identify key biomarkers associated with spoilage progression in refrigerated goose meat, Pearson correlation analysis was conducted between TVB-N and 211 differential metabolites. The results demonstrated that 49 metabolites exhibited statistically significant correlations with TVB-N (*p* < 0.05), including 41 positively correlated metabolites and 8 negatively correlated metabolites.

Notably, among the 30 screened biomarkers, 5-hydroxy-2′-deoxyuridine, riboflavin, xanthine, and N-(2-hydroxyethyl) stearamide exhibited significant positive correlations with TVB-N (*p* < 0.05) (Figure 4B), suggesting their potential roles as spoilage indicators in goose meat. 5-hydroxy-2′-deoxyuridine (oh5dU), the main product of DNA oxidation, directly reflects the accumulation of oxidative DNA damage during cold storage [35]. Xanthine (Xt), the xanthine oxidase-catalyzed oxidation product of hypoxanthine (Hx), has been established as a critical spoilage biomarker in chicken and fish species [36,37]. This study demonstrated that xanthine accumulation in refrigerated goose meat exhibits a time-dependent increase, consistent with its role as a spoilage indicator of meat. Riboflavin (vitamin B2), a redox-active coenzyme, exhibited a time-dependent increase in content during 0–7 days of refrigerated storage (Table S1). Similarly, Miao et al. (2024) reported that both (+)-riboflavin and (−)-riboflavin concentrations consistently rose over 0–9 days of refrigerated storage (4 °C) in goose meat [38]. The accumulation of xanthine and riboflavin may signify intensified nucleotide catabolism and oxidative reactions driven by microbial metabolism or autolytic enzymes [39,40]. Conversely, the negative correlations of glutathione oxidized (GSSG), inosine 5′-monophosphate (IMP), and guanine with TVB-N highlight their depletion as critical markers of freshness loss (Figure 4B). GSSG, the oxidized form of glutathione (GSH), serves as a critical biomarker and regulatory factor in maintaining cellular redox homeostasis. The observed GSSG reduction in our study may result from antioxidant system activation, where increased antioxidant demand during oxidative stress enhances NADPH-dependent glutathione reductase-mediated conversion of GSSG to GSH [41]. Such a decline in GSSG is likely to reflect impaired redox balance, thereby accelerating meat quality deterioration, including discoloration and off-flavor development. This observation is consistent with the findings of Michiels et al. (2014), who reported that oxidative stress triggered by free-range husbandry and feed restriction significantly impaired broiler meat quality, as characterized by increased muscle lipid oxidation and accelerated meat color deterioration [42]. IMP, a pivotal umami-enhancing compound in meat, showed a significant negative correlation with TVB-N, indicating its rapid depletion is a key indicator of progressive freshness loss during refrigeration. IMP undergoes rapid enzymatic degradation in postmortem muscles, being sequentially converted into hypoxanthine riboside (HxR) and hypoxanthine (Hx), both of which have a bitter taste [4]. In this study, the IMP content exhibited no significant difference within the first 5 days but decreased markedly by day 7 (Table S2), suggesting that day 5 may present a critical threshold for flavor preservation in goose meat during 4 °C refrigeration. Guo et al. (2024) detected changes in volatile organic compounds (VOCs) and bacterial community structure in goose meat stored at 4 °C, revealing that day 3 marked the maximum shelf life based on microbial safety [6]. The apparent discrepancy between the flavor threshold (day 5) and the safety threshold (day 3) in goose meat likely arises from distinct deterioration drivers. Microbial activity dominates early spoilage and defines shelf life, while endogenous enzymes progressively deplete IMP and govern flavor degradation. Sequential degradation occurs as microbial proliferation likely precedes nucleotide catabolism, based on the findings of Guo et al. (2024), who identified day 3 as the safety threshold [6]. This sequential pattern suggests that day 3 may represent the safety threshold, and day 5 may represent the sensory threshold in goose meat. However, as microbial activity was not directly assessed in this study, the attribution of early spoilage primarily to microbial processes and later flavor loss to endogenous enzymatic activity, although consistent with Guo et al.’s findings [6] and established degradation pathways, remains a plausible hypothesis requiring future validation through integrated microbial and biochemical analyses. Similar progressive declines in IMP content have been documented in chicken. Wen et al. (2020) [43] observed this decline in chicken meat stored at 4 °C for 10 days. Critically, based on the observed variation trends of IMP and other nucleotides, Wen et al. determined that the maximum shelf life of refrigerated chicken reaches 6 days, yet recommended consumption within 4 days to ensure optimal flavor [43]. Ju et al. (2025) further demonstrated that chicken meat stored at 4 °C exhibited detectable flavor deterioration by day 8, with complete spoilage occurring by day 10, as evidenced by TVB-N levels exceeding the freshness threshold for poultry meat [8]. Critically, a recent comprehensive study evaluating bacteriological quality and physicochemical properties supports these findings, establishing the maximum freshness limit and shelf life for refrigerated chicken meat at 3–5 days [44]. This timeframe aligns closely with the sensory threshold identified in the present goose study (day 5) and the safety thresholds reported elsewhere (day 3 in goose [6]; microbial growth thresholds in chicken [44]). The observed difference in critical sensory thresholds between goose and chicken may stem from inherent species-specific factors, such as muscle composition, variations in initial IMP content, or microbial ecology during refrigerated storage. Guanine depletion in refrigerated goose meat likely stems from the enzymatic conversion of guanine to xanthine, mediated by microbial or endogenous deaminases. This hypothesis is supported by the progressive accumulation of xanthine during storage (Table S2). Collectively, the accumulation of DNA damage/lipid oxidation markers and depletion of antioxidant/flavor compounds serve as robust biochemical indicators of spoilage progression in refrigerated goose meat, with IMP depletion defining the critical flavor threshold at day 5.

### 3.5. Evolution of the Differential Metabolites During the Refrigeration of Goose Meat

#### 3.5.1. Animo Acids and Their Metabolomics

**Figure 5:**
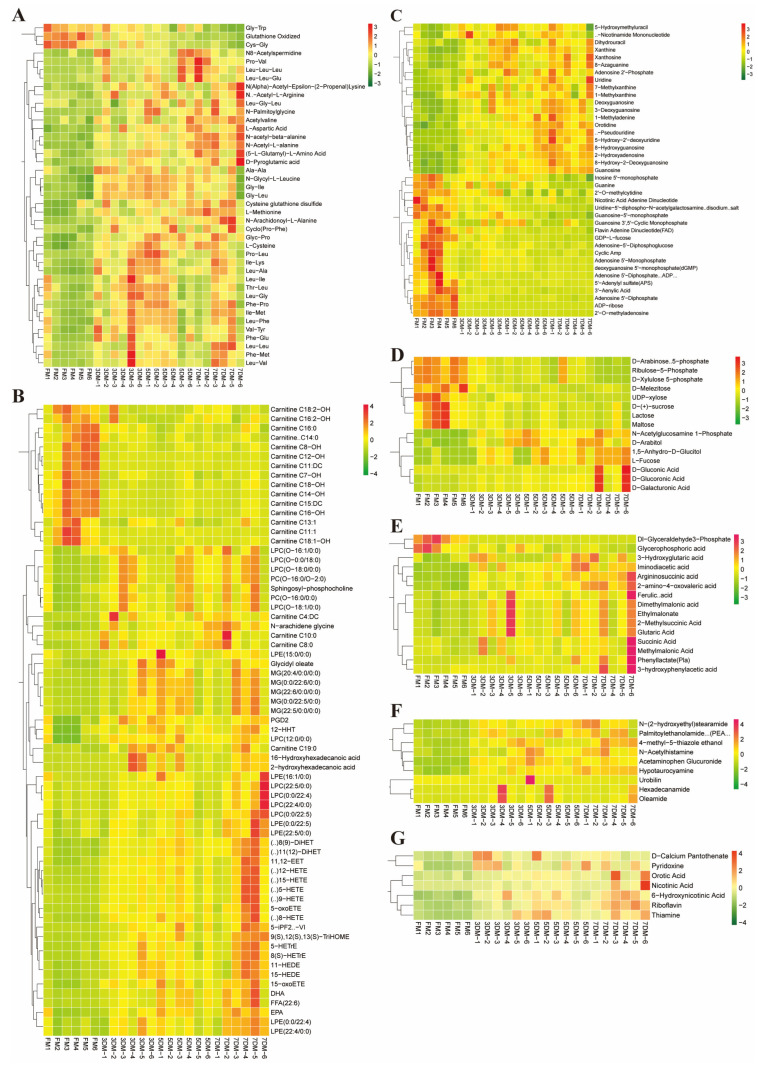
Heat map illustrating the expression pattern of differential metabolites among FM, 3DM, 5DM, and 7DM. The expression profiles of (**A**) differential amino acids and their metabolites, (**B**) nucleotides and their metabolites, (**C**) lipid metabolites, (**D**) carboxylic acids and their metabolites, (**E**) organic acids and their metabolites, (**F**) alcohol and amines, (**G**) coenzymes and vitamins.

During refrigeration, meat spoilage progresses through increased proteolysis and microbial growth, generating peptides and free amino acids [45]. In this study, 188 amino acids and metabolites were screened in chilled goose meat. Among these, 41 were identified as differential metabolites, primarily free amino acids (FAA) and small peptides, demonstrating significant protein degradation over time (Figure 5A). Of note, the amount of oxidized glutathione (GSSG), Cys-Gly, and Gly-Trp sustainably decreased with prolonged refrigeration time. GSSG is known to have antioxidant activity, thereby preventing damage to cellular components caused by ROS (e.g., free radicals, peroxides, and lipid peroxides) [46]. The decrease in GSSH level with aging indicated the gradual incapacitation of the antioxidant defense system in refrigerated goose meat, potentially accelerating meat spoilage. This is consistent with findings that compromised antioxidant defense system exacerbates oxidative reactions, inducing myofibrillar protein oxidation, structural changes (cross-linking and aggregation), and consequent impairment of muscle water-holding capacity [47]. FAA are mainly responsible for the tastes of meat, such as umami, sweetness, saltiness, and bitterness [48]. Our data showed that the majority of FAA, such as N-Arachidonoyl-L-alanine, Acetylvaline, N-acetyl-beta-alanine, and D-Pyroglutamic acid, increased progressively during refrigeration. This aligns with chicken breast studies showing elevated FAA levels in early chilling (0–6 days at 2 °C), followed by a decline [45]. Such dynamic FAA profiles indicate significant flavor alterations in refrigerated goose meat. Additionally, small peptides (e.g., Ala-Ala, Gly-Ile, Leu-Ile, Pro-Leu, Val-Tyr) peaked on days 3–5 before decreasing by day 7. This transient accumulation likely reflects calpain-mediated proteolysis [49], consistent with reported calpain degradation patterns in goose muscle at 5 °C [50]. Together, proteolytic events collectively degrade flavor stability in refrigerated goose meat, as their peptide and FAA products disrupt its native balance within 7 days.

#### 3.5.2. Nucleotides and Their Metabolites

Nucleotides critically influence meat flavor perception alongside free amino acids. These low–molecular-weight, water-soluble nitrogenous compounds, such as adenosine triphosphate (ATP), serves as microbial substrates and undergo spoilage-related enzymatic transformations. In chilled or frozen meat, ATP is rapidly degraded by endogenous and microbial enzymes into inosine monophosphate, hypoxanthine, and other breakdown products, directly altering flavor profiles and freshness indicators [4]. We identified 91 nucleotide compounds, of which 39 showed significant metabolic shifts during refrigeration. Twenty metabolites accumulated progressively, likely from endogenous enzymatic degradation in muscle tissue. Conversely, 19 nucleotides decreased substantially after day 3 (Figure 5B), including key umami-enhancing compounds: inosine-5′-monophosphate (IMP), adenosine-5′-monophosphate (AMP), adenosine-5′-diphosphate (ADP), and guanosine-5′-monophosphate (GMP). IMP holds particular significance as the primary ATP degradation product and is recognized as the principal umami compound responsible for the desirable fresh meat flavor perception [8,51]. However, its inherent instability in postmortem muscle leads to rapid enzymatic degradation. The well-characterized postmortem nucleotide degradation cascade involves the sequential conversion of ATP through ADP, AMP, IMP, inosine (HxR), and hypoxanthine (Hx) via ATPase, myokinase, and nucleosidase/phosphatase activities [52]. Critically, the enzymatic degradation of IMP into HxR and subsequently Hx is associated with the development of bitter off-flavors during storage [43]. Our findings revealed marked IMP depletion from day 5 to day 7 of storage, accompanied by gradual increases in HxR and Hx levels. This significant reduction in IMP by day 7 directly corresponds to the loss of the key umami taste and the emergence of undesirable flavors, as highlighted by its correlation with TVB-N in Section 3.4. Moreover, the increase in Xt paralleled the progressive decline in IMP content during refrigerated storage of goose meat, suggesting that Xt also serves as a spoilage indicator in goose. Collectively, from the perspective of purine metabolism, these metabolic shifts suggest that while 4 °C refrigeration progressively diminishes the freshness and umami characteristics of goose meat, a 5-day storage window may represent the optimal shelf life for maintaining desirable flavor. This 5-day optimal shelf life for goose meat under refrigeration aligns with findings in chilled duck meat, where the untreated group reached the microbial spoilage threshold by day 5, suggesting 5 days may be a critical freshness limit for refrigerated duck [53].

#### 3.5.3. Lipid Metabolites

Lipid dynamics during storage serve as critical indicators of meat quality, with environmental factors such as temperature, moisture, and light exposure significantly influencing lipid stability [54]. Lipid oxidation constitutes a primary mechanism of quality deterioration, progressively compromising sensory attributes, flavor profiles, and nutritional value through oxidative damage exacerbated by depletion of the endogenous antioxidant system [55]. In the present study, we identified 67 differential lipid metabolites. Notably, 15 acylcarnitines, including Carnitine C18:1-OH, Carnitine C18:2-OH, and Carnitine C16-OH, exhibited progressive depletion correlated with refrigeration time (Figure 5C). This finding aligns with Zhang et al.’s observation in sheep, where postmortem chilled aging at 4 °C similarly reduced fatty acylcarnitine levels [56]. As essential transporters facilitating mitochondrial fatty acid β-oxidation, the observed decline in acylcarnitines signifies enhanced fatty acid catabolism during refrigerated storage.

In contrast to acylcarnitines, most free fatty acids (FFA), including glycerophospholipids, monoglycerides, and polyunsaturated fatty acids (PUFA), increased during the 7-day refrigeration (Figure 5C). This aligns with Williamson et al. [57], who documented substantial FFA accumulation in postmortem beef aged at 4 °C. FFAs originate from lipolysis, where endogenous and microbial enzymes hydrolyze phospholipids and triacylglycerols [58]. Our data indicate similar mechanisms in refrigerated goose meat, with phospholipase-mediated conversion of phospholipids to FFA and lysophospholipids [56]. This is supported by concurrent increases in lysophosphatidylcholine (LPC) and lysophosphatidylethanolamine (LPE) (Table S2). The high unsaturated fatty acid (UFA) content in goose meat, particularly its rich PUFA content, predisposes it to oxidative deterioration during storage, since these compounds are highly susceptible to oxidation, which presents a key factor in quality deterioration [2]. Our analysis revealed that the progressive accumulation of ω-3 PUFAs, notably docosahexaenoic acid (DHA) and eicosapentaenoic acid (EPA), correlates with sensory defects, including tallowy flavor, bitterness, and metallic off-notes [59]. Mechanistically, this process generates volatile aldehydes, ketones, and esters responsible for off-flavors [60]. Furthermore, the significant increase in N-arachidene glycine, a validated biomarker of oxidative stress [61], confirms pronounced lipid oxidation in refrigerated goose meat, directly linking oxidative pathways to sensory deterioration.

#### 3.5.4. Carboxylic Acids and Organic Acids

Carbohydrate metabolism critically influences postmortem meat quality, with its decomposition products serving as key indicators of storage-induced deterioration [62]. Our metabolomic analysis identified 15 differential carboxylic acids and derivatives, systematically categorized into sugars, sugar alcohols, sugar acids, and sugar phosphates. Sugars and sugar phosphates were progressively depleted during refrigeration, while sugar alcohols and sugar acids accumulated reciprocally (Figure 5D, Table S2). This sugar depletion primarily reflects microbial consumption and endogenous enzymatic activity [63]. Consistent with poultry studies, the reduction in the saccharide content we observed mirrored the pattern seen in chicken, where saccharides are completely depleted within 4 days at 4 °C [43]. Notably, sugar phosphates are glycolytic intermediates generated during muscle glycogen conversion to lactate. While their specific impact on poultry quality requires further study, our observed reduction parallels findings in ovine meat, where diminished phosphate-sugar levels correlated with reduced color stability [61]. This suggests refrigeration duration may progressively influence goose meat chromaticity via phosphate-sugar modulation.

Organic acids, key intermediates in carbohydrate metabolism, significantly impact meat’s nutritional value, flavor, and sensory quality [64]. Our metabolomic profiling identified 88 organic acids, among which 15 exhibited progressive accumulation during storage (Figure 5E, Table S2). This pattern indicates accelerated post-mortem carbohydrate metabolism under refrigeration. While moderate levels of these acids contribute positively to flavor complexity, excessive accumulation, particularly between days 3–7, correlates with marked freshness decline in goose meat [65]. Mechanistically, this sensory deterioration may stem from competitive binding to umami receptors by accumulated acids [66], effectively masking desirable taste perception. Notably, the sustained rise of succinic acid, a well-established microbial metabolite derived from glucose catabolism, serves as a key indicator of proliferating microbial activity [67]. This acid profile evolution highlights the complex interplay between microbial activity and endogenous enzymes in determining refrigerated meat’s organoleptic fate [68].

#### 3.5.5. Bioamines, Coenzymes and Vitamins

Bioamines, low molecular weight organic bases formed by enzymatic decarboxylation of amino acids, serve as established biomarkers for meat spoilage [69]. In poultry, histamine, tyramine, and polyamines predominate, exhibiting physiological importance but potential toxicity at elevated levels, with histamine posing particular health risks [70]. Herein, we identified seven differential bioamines, including N-acetylhistamine, N-(2-hydroxyethyl) stearamide, and Hypotaurocyamine, exhibited significant time-dependent accumulation during refrigerated storage (Figure 5F, Table S2). Consistently, chicken meat displayed accelerated bioamine generation, with histamine, tyramine, and N-acetylcadaverine concentrations initiating ascent by day 2 and demonstrating marked accumulation by day 4 at 4 °C [43]. This accelerated bioamine generation in chicken likely reflects species-specific decarboxylase activation kinetics. In parallel, bioamine accumulation in goose meat predominantly manifests progressive microbial decarboxylase activity and endogenous enzyme action [71], collectively revealing distinct spoilage progression pathways across poultry species.

Among the 15 coenzymes and vitamins examined here, 7 showed significant changes during refrigeration. Specifically, the contents of D-calcium pantothenate (vitamin B5) and pyridoxine (vitamin B6) significantly increased during refrigeration and reached their peak on day 3. Conversely, thiamine (vitamin B1), riboflavin (vitamin B2), nicotinic acid (vitamin B3), orotic acid, and 6-hydroxynicotinic acid demonstrated progressive accumulation (Figure 5G). As a group of organic cofactors related to enzyme-catalyzed REDOX, group transfer, and isomerization reactions, coenzyme and vitamins play an important role in carbohydrate, lipid, and amino acid metabolisms. The increase in these coenzyme and vitamins indicated a gradual decrease in the physiological activity of the muscle cells, leading to their hydrolysis from the enzyme-catalyzed system [72], especially during the first three days of refrigeration.

#### 3.5.6. The Potential Metabolism Process of Goose Meat During Refrigeration

**Figure 6:**
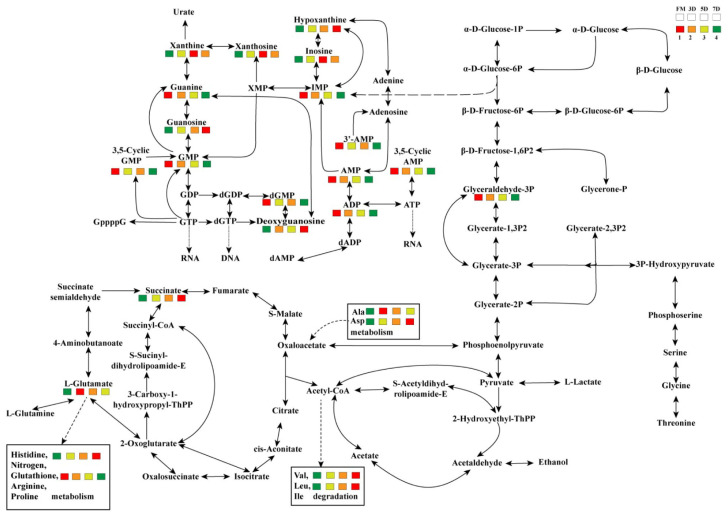
The metabolic pathways of potential metabolic biomarkers in goose breast meat during chilling storage. The heat map represents the relative content of metabolites, decreasing gradually from left to right. The bar represents groups of FM, 3DM, 5DM, and 7DM from left to right. AMP, adenosine monophosphate; ATP, adenosine triphosphate; RNA, ribonucleic acid; ADP, adenosine diphosphate; IMP, inosine monophosphate; XMP, xanthosine monophosphate; dGMP, deoxyguanosine monophosphate; dAMP, deoxyadenosine monophosphate; dGDP, deoxyguanosine diphosphate; dGTP, deoxyguanosine triphosphate; DNA, deoxyribonucleic acid; GMP, guanosine monophosphate; GDP, guanosine diphosphate; GTP, guanosine triphosphate; Ala, alanine; Asp, aspartate; Val, valine; Ile, isoleucine.

To elucidate the metabolic dynamics underlying quality changes in refrigerated goose meat, we systematically investigated the pathway networks and transformation patterns of characteristic metabolites. Focused analysis was conducted on purine, carbohydrate, and amino acid metabolic pathways, which represent critical biochemical routes intimately associated with meat quality attributes and flavor development. Nucleotide metabolism predominantly involved purine and pyrimidine pathways. Notably, purine metabolism represents one of the most significant biochemical transformations in postmortem muscle tissue and serves as a reliable indicator for meat freshness evaluation [8]. As illustrated in Figure 6, temporal variations were observed in the concentrations of IMP, AMP, ADP, GMP, guanine, xanthine, and hypoxanthine within the purine metabolic network. These dynamic changes suggest that monitoring purine metabolite profiles could provide critical insights into the biochemical mechanisms driving quality deterioration during refrigerated storage. Carbohydrate metabolism analysis revealed critical changes in glycolytic pathways and TCA cycle components in goose meat during refrigerated storage at 4 °C. Glyceraldehyde-3P, a pivotal glycolytic intermediate, showed a significant decrease over the refrigerated storage duration, indicating its potential as a quality biomarker for postmortem aging assessment [38]. The observed decline in this key metabolite suggests progressive attenuation of glycolytic flux during refrigeration. Amino acid metabolic profiling identified substantial variations in Val, Leu, Ala, and Asp concentrations. Of note, histidine, a precursor substance to histamine, was significantly increased with the refrigeration time, implying a corresponding increase in the histamine content. Given that histidine and histamine are both essential biomarkers for freshness and spoilage in poultry meat [73], our findings suggested that N-Acetylhistidine, identified among the top-ranked candidate biomarkers through RFR analysis, emerges as a novel diagnostic biomarker for goose meat quality evaluation during refrigerated storage. Overall, our data provided a molecular framework for understanding the spoilage continuum in refrigerated goose breast meat, revealing that quality deterioration results from synergistic interactions between multiple biochemical pathways rather than isolated metabolic events.

#### 3.5.7. Practical Implications and Future Perspectives

The identified low-molecular-weight purine metabolites demonstrate significant translational potential across the food supply chain. For industry applications, hypoxanthine (Hx) exhibits a strong correlation with TVB-N, enabling rapid quality screening through paper-based biosensors integrated with smartphone analysis. This approach reduces TVB-N testing time from 6 h to 10 min [74], providing slaughterhouses with field-deployable tools. Concurrently, our observed IMP depletion kinetics in goose meat may offer industry a quantifiable freshness indicator for automated grading systems when integrated with MXene-Au@Pt biosensors [75]. For consumer empowerment, riboflavin’s concentration-dependent fluorescence properties provide a non-destructive spoilage monitoring mechanism. The >90% fluorescence intensity reduction at pH 5 during milk spoilage provides a quantifiable threshold for intelligent packaging [76]. Such technology could trigger visible indicators when metabolites exceed critical thresholds, empowering consumers to make informed purchasing decisions. Moreover, the dynamic profile of purine metabolites, such as Xt accumulation and IMP depletion at Day 5 in refrigerated goose meat, may serve as a digital biomarker for predictive shelf-life models.

## 4. Conclusions

In summary, we provided a widely targeted metabolomic approach based on UHPLC-MS/MS for systematically elucidating the evolutionary trajectories of characteristic metabolites and metabolic pathways of goose meat during refrigerated storage. A total of 211 differential metabolites were identified, with 30 potential biomarkers prioritized via random forest regression. Seven biomarkers, including 5-hydroxy-2′-deoxyuridine, N-(2-hydroxyethyl) stearamide, riboflavin, xanthine, oxidized glutathione, inosine 5′-monophosphate, and guanine, exhibited strong correlations with TVB-N. Our analysis further revealed that purine degradation, amino acid metabolism, and sugar-driven pathways are pivotal in goose meat deterioration. By integrating physicochemical and metabolic profiling, we identified Day 5 as the critical threshold for quality deterioration during 4 °C storage.

The identified biomarker dynamics provide a mechanistic foundation for developing practical monitoring tools. Xanthine, amenable to rapid detection via lateral flow assays, and IMP, serving as a quantifiable freshness indicator detectable with advanced biosensors, represent key purine degradation products. Riboflavin’s intrinsic fluorescence offers potential for cost-effective, non-destructive indicators in intelligent packaging. Future studies will focus on translating these findings into field-deployable tools (e.g., biosensing platforms for IMP/Hx, test strips for xanthine, packaging indicators for riboflavin) and integrating the dynamic profiles of multiple purine metabolites with storage conditions to build robust predictive shelf-life models leveraging these digital biomarkers. Future studies will focus on developing portable detection kits (such as test strips) for these biomarkers, and combining multiple metabolite changes with storage conditions to build shelf-life prediction tools.

## Figures and Tables

**Table 1:** Ingredient and nutrient levels of the commercial diets for geese (1~70 d).

Item	1~28 d	29~70 d
Ingredients, %		
Corn	64.0	61.5
Soybean meal	27.0	13.6
Fish meal	3.0	3.0
Alfalfa meal	2.0	16.0
Soybean oil	0.0	2.0
Dicalcium phosphate	1.8	1.9
Limestone	0.9	0.8
Salt	0.3	0.2
Vitamin and trace mineral mix	1.0	1.0
Nutrient levels		
Apparent ME, KJ/kg	11.2	10.85
Crude protein, %	18.0	15.75
Calcium, %	0.8	0.8
Total phosphorus, %	0.42	0.37
Crude fiber, %	4.85	6.0
Lys, %	0.9	0.65
Met, %	0.4	0.33
Sulphur-amino acid, %	0.79	0.56
Trp, %	0.17	0.13
Thr, %	0.8	0.8
Na, %	0.3	0.3
Cl, %	0.25	0.24

Vitamin and trace mineral mix supplied the following per kilogram of total diet: vitamin A, 20,000 IU; vitamin D3, 4500 IU; vitamin E, 300 IU; vitamin K3, 20 mg; vitamin B1, 10 mg; vitamin B2, 120 mg; vitamin B6, 20 mg; vitamin B12, 0.2 mg; nicotinic acid, 600 mg; pantothenic acid, 180 mg; folic acid, 10 mg; folate, 10 mg; biotin, 0.8 mg; choline, 7 g; Fe, 1.2 g; Cu, 0.2 g; Mn, 1.9 g; Zn, 1.8 g; I, 10 mg, Se, 6 mg. ME: metabolic energy.

## Data Availability

The original contributions presented in the study are included in the article/Supplementary Materials. Further inquiries can be directed to the corresponding authors.

## Supplementary Materials

The following supporting information can be downloaded at: https://www.mdpi.com/article/10.3390/foods14172950/s1, Figure S1: The score plots and model validation of OPLS-DA pairwise comparisons; Figure S2: Profile analysis of 30 potential metabolic biomarkers identified by random forest regression. Each group (FM, 3DM, 5DM, 7DM) contains 6 replicates; Table S1: Identified metabolites in goose meat during refrigerated storage (n = 748); Table S2: Differential metabolites in goose meat during refrigerated storage (n = 211); Table S3: The 30 potential biomarkers identified by random forest and validated by the Kruskal–Wallis test.
