# Supplementary figures and images for: UHPLC-MS/MS-Based Metabolomics Identifies Freshness Biomarkers and Temporal Spoilage Threshold in Refrigerated Goose Meat
Source: Foods. 2025 Aug 24;14(17):2950. doi: 10.3390/foods14172950 (PMC12428737; doi:10.3390/foods14172950)

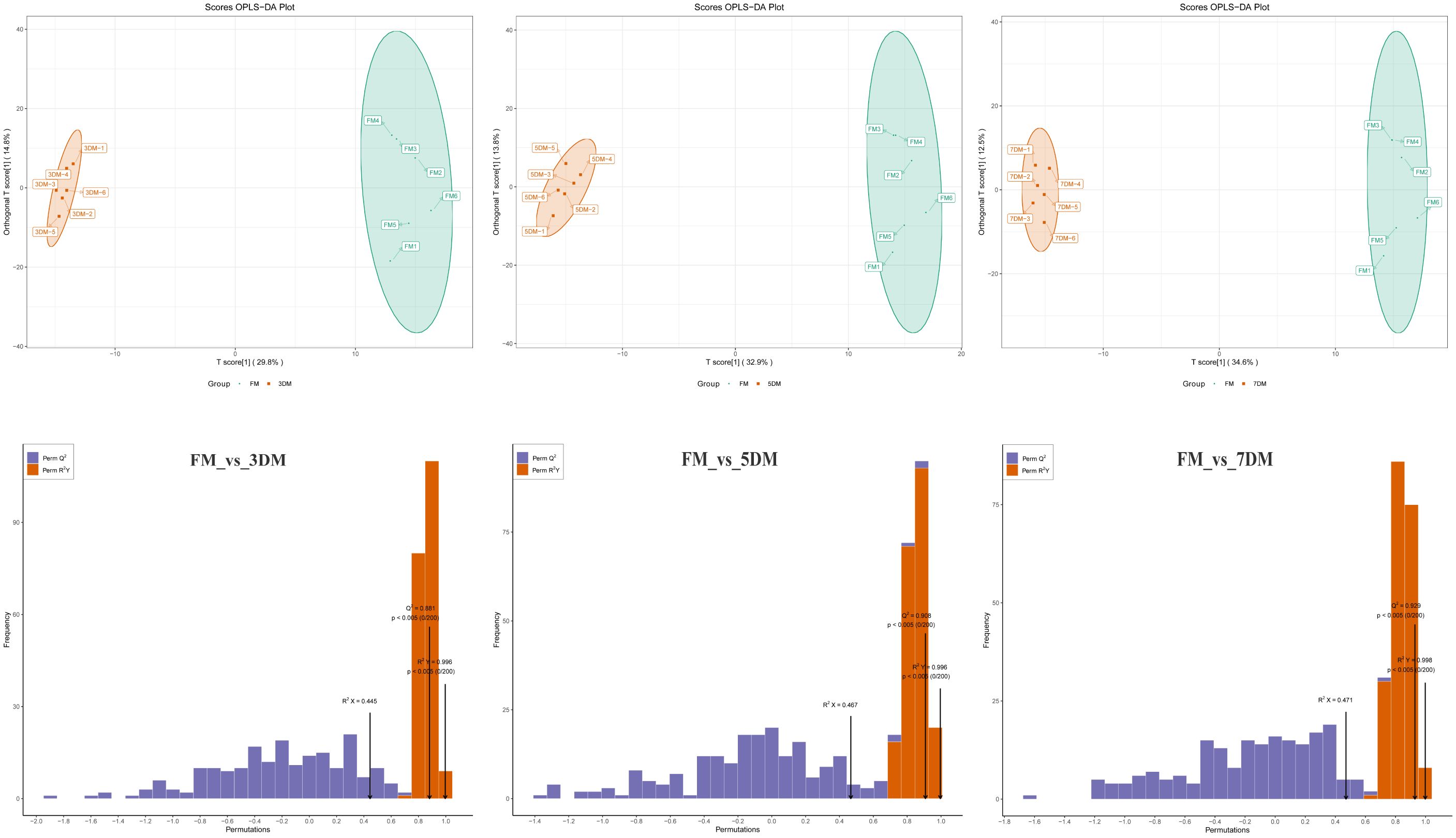

Supplement: Supplementary file 1 [file foods-14-02950-s001.zip › Figure S1..tif]

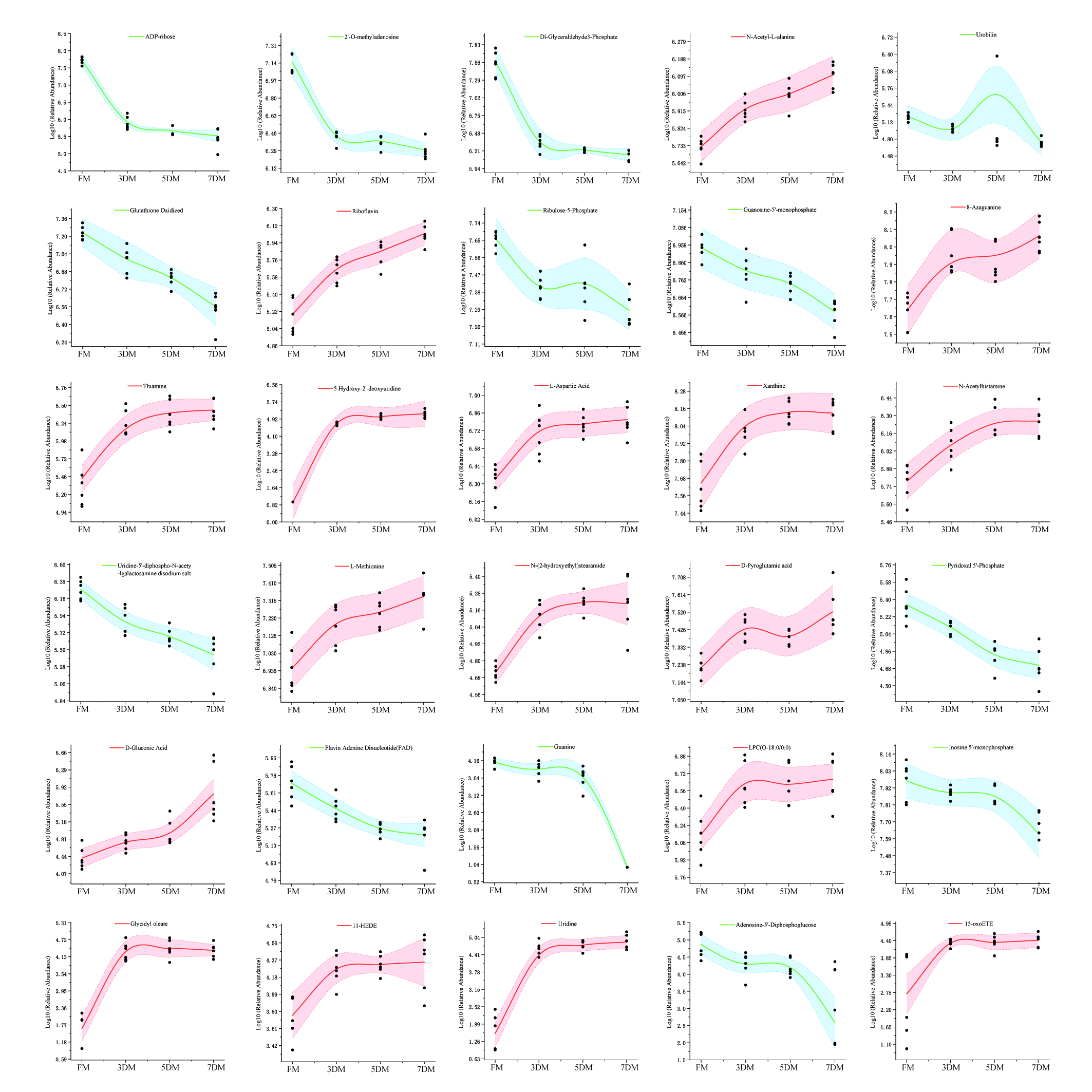

Supplement: Supplementary file 1 [file foods-14-02950-s001.zip › Figure S2..tif]
